# Clinical Analysis for Diagnosing Autism in Children Under Two: A Case Report

**DOI:** 10.7759/cureus.67888

**Published:** 2024-08-27

**Authors:** Maria Fernandez, Augusta Soyele, Toritseju Arenyeka, Kiran Hashmi, Ruvarashe Mupedziswa

**Affiliations:** 1 Pediatric Medicine, Florida International University, Miami, USA; 2 Neurology, Florida International University, Miami, USA; 3 Surgery, Florida International University, Miami, USA; 4 Internal Medicine, Florida International University, Miami, USA; 5 Internal Medicine, Bassett Healthcare Center, New York, USA

**Keywords:** macrocephaly, premature infants, mchat-r/f, mchat, autism and chromosome 19, autism and hand wringing, autism and genes, autism and communication, autism screening, : autism spectrum disorder (asd)

## Abstract

Autism spectrum disorder (ASD) is a complex neurodevelopmental condition with rising prevalence, necessitating early diagnosis and intervention. This case report examines the clinical diagnosis approach of ASD in children under two years, emphasizing motor developmental delay, chromosome 19 mutations, prematurity, macrocephaly, and false-negative Modified Checklist for Autism in Toddlers (MCHAT) results. This study identifies gross motor delays as a potential key indicator in the diagnosis of ASD, as all five cases (Patients A, B, C, D, and E) were observed to have such deficits. Two cases (Patients A and B) initially had negative MCHAT results but were later diagnosed with ASD. Patients C and E both had a chromosome 19 abnormality. Patient E had macrocephaly and an amino acid metabolism disorder. ASD atypical behaviors like hand flapping, wringing, and twirling were also noted. Our systematic review validated the key findings presented in this study, unveiling a consistent pattern throughout the existing literature about ASD diagnoses and the associated misconceptions. These cases highlight the significance of early motor delay, genetic factors, and the limitations of MCHAT in early ASD diagnosis. This case report underscores the need for improved screening tools, genetic investigations, and comprehensive assessments to enhance early detection and intervention for ASD. Early identification and personalized interventions hold a promise to improve the outcomes and quality of life for children with autism.

## Introduction

Autism spectrum disorder (ASD) is a multifaceted neurodevelopmental condition characterized by a range of social, communication, and behavioral challenges. ASD has emerged as a pressing concern in modern healthcare, with a consistent rise in diagnosed cases over recent years, leading to an estimated increase in prevalence from 1 in 44 children in 2021 to 1 in 36 in 2023 (CDC, 2023). Autism is often referred to as a spectrum because it encompasses a diverse range of individuals with varying levels of impairment and unique strengths. Some individuals with autism have significant communication difficulties and intellectual disabilities, while others may have milder symptoms and excel in certain areas. The core symptoms of autism typically manifest in early childhood. They may include difficulty making eye contact, understanding social cues, delayed speech development, repetitive or restricted behaviors, repetitive hand-flapping, and intense focus on specific interests. While the average age of ASD diagnosis in the United States is around four years old, recent advances in research and clinical practice have paved the way for the possibility of diagnosing ASD in children under two years of age [1]. Early diagnosis and intervention are pivotal for improving individuals' long-term outcomes and quality of life with ASD. Additionally, emerging research has emphasized the significance of identifying potential indicators of autism in infants under the age of two [2].

Several scholarly articles have contributed valuable insights to this evolving field. An extensive review spanning databases from Embase, Medline, Scopus, and others from 2000 to 2022 [3] provided insights into the behavioral patterns of infants during their initial six months. This review underscored certain behaviors, such as general attention, attention towards social stimuli, and motor activities, as potential indicators of subsequent ASD diagnoses. Notably, diminished attention measures and asymmetric visual tracking were predominantly observed in infants who were later diagnosed with ASD. From 2012 to 2016, a descriptive study of 50 consecutive cases clinically diagnosed with ASD revealed social interaction deficits as the primary consultation cause, with ~86.4% of participants exhibiting language impairments or atypical language development. This article showed that the core features of ASD encompass deficits in social communication and the presence of repetitive behaviors, stereotyped actions, or speech, along with an insistence on sameness and unusual sensory responses [4].

Recent reviews and studies have further refined the diagnostic criteria for ASD, highlighting the importance of factors such as social communication, behavioral rigidity, and learning disabilities. For instance, an observational study on neonatal neurobehavior indicated a pronounced vulnerability in attention within the first three months, which might be a precursor to emerging ASD [5]. Interventions like the Modified Checklist for Autism in Toddlers have expedited early diagnoses. Between 2013 and 2016, the median diagnostic age dropped significantly compared to the previous years. Recognizing early motor delays, often considered a prodrome of ASD, alongside parent-reported concerns, has also aided early detection [6].

Additionally, emerging research on infants at high ASD risk has furnished invaluable insights into the disorder's early development. While social communication abnormalities typically manifest during the second year, other "prodromal features" like motor and sensory irregularities emerge earlier [7]. Lastly, the *Prospective Study of Infant Siblings of Children with Autism* underscores the significance of studying siblings of children with autism to identify early markers and risk factors. This comprehensive background aims to set the stage for a case report that explores the clinical approach to diagnosing autism in children under two on global motor developmental delay as a pivotal early indicator.

## Case presentation

Patient A

A series of milestones, challenges, and diagnoses marked patient A's developmental journey. At six months, the patient exhibited congenital torticollis and preferred to sleep on his right side; this was resolved after attending physical therapy. By his nine-month checkup, Patient A exhibited progress in some areas, such as feeding well, bathing daily, crawling, imitating speech sounds, pulling himself from a sitting position, and standing while holding on. However, he had not yet achieved the developmental milestone of using a pincer grasp, which is an important fine motor skill. These continued developmental challenges prompted the referral to a neurologist to investigate his delayed fine motor skills. At Patient A's one-year checkup, further progress was evident, including his ability to bang two cubes together, demonstrate a pincer grasp, play with a ball, engage in *Pat-a-cake*, drink from a cup, say, *Dada* and *Mama*, stand-alone and walk while holding furniture. While these developments were promising, there were concerns about fine motor skills, such as not scribbling with a pencil and not feeding himself with minimal assistance. The 15-month checkup showed that Patient A continued to make progress by drinking from a cup, feeding himself, scribbling spontaneously, stacking a tower of two cubes, using jargon, using a spoon, and walking independently with no issues in gait. However, fine motor challenges persisted.

In 2022, the patient exhibited a negative MCHAT. At the 18-month checkup, Patient A demonstrated the ability to dump raisins from a bottle, follow directions (2 out of 3 times), point to body parts, help with simple tasks, and stack a tower of four cubes. The patient also exhibited echolalia; when phrases were said, the patient would mirror the last words spoken. Throughout his physical examinations, the patient always exhibited resistance to being touched. He giggled and flinched when being examined, and at times, he would also exaggeratedly cry. One distinct red flag that was noted by the physician was his exaggerated stranger anxiety, which is a notable sign of autism. Fine motor difficulties remained a concern. During Patient A's third-year checkup, he received a significant diagnosis of ASD at level 2, indicating substantial challenges in social communication and the presence of repetitive behaviors.

Additionally, he was diagnosed with an expressive language disorder, developmental delay, and an unspecified sensory disorder. Behavioral characteristics of ASD, such as flapping fingers, were observed, leading to the need for an Individualized Education Program (IEP). Patient A's selective eating habits and sleep patterns were also noted during this assessment. This case report explores Patient A's complex developmental journey, highlighting a combination of developmental milestones, challenges, and complex diagnoses. When the patient was diagnosed, the parents promptly enrolled him in occupational, speech, behavioral, and physical therapy therapies. Throughout the subsequent visits, the patient exhibited behavioral improvement; his stranger anxiety was not as severe, and he was able to converse with the physician and answer questions regarding his colors and shapes correctly. The parent even attested to a significant improvement in his behavior with sustained therapy.

Patient B

Patient B is a three-year-old boy who presents with signs of suspected ASD despite no official ASD diagnosis. At his four-month visit, the patient presented with blunt head trauma secondary to a fall from bed with no loss of consciousness (LOC). At his six-month visit, the patient met all his milestones. He babbled, squealed, sat briefly, turned to voices, reached for toys, and bore weight on one leg. At his nine-month visit, the patient also met all his milestones. At his one-year visit, the patient was able to bang two cubes together, drink from a cup, pincer grasp, say *mama and dada*, play ball, and *Pat a Cake*. However, the patient could not walk well and presented to the clinic with gross motor developmental delay. At his 15-month visit, the patient could not walk independently; physical therapy was referred, but there was a failure to follow up. At his 18-month visit, the patient presented with recurring gross motor developmental delay, and the parents refused his 18-month vaccines. The parents met with the Department of Health for exemption due to vaccination refusal. The patient has started walking, although his gait is unsteady. At his two-year visit, the patient presented with a negative MCHAT and was upset by loud noise but had no issues with a vacuum. The patient also presents with exaggerated stranger anxiety and was referred for a neurology consult because he has hand-flapping and temper tantrums.

Additionally, the patient had difficulty controlling behaviors. At his most recent visit in 2023, he presented with expressive speech delay, and a referral to speech therapy was given. The patient continued to be referred to a neurologist in hopes of confirming the diagnosis, but there was no compliance. Patient B also has a younger brother who was 13 months old and was being observed due to similar atypical movements, such as hand twirling, during physical examination. In addition to the hand twirling, good eye contact was also observed. 

Patient C

Patient C is a six-year-old boy who was born prematurely in twin gestation. The patient also presented with a past medical history of infantile spasms that was treated with ACTH. Upon examination, the physician noted that the patient was severely hypotonic and was lying in a frog position. The physician had concerns as to where the patients would be able to ambulate independently. At his 15-month visit, the patient presented with developmental speech delay; he did not say *mama and dada*; he could not walk alone, stack two cubes, use jargon, drink from a cup, or feed himself. The patient also had an expressive language delay and received speech, physical, and occupational therapy. At the 18-month visit, the patient presented with continued developmental delay, and chromosome 19 abnormality was detected. He could not follow 2 out of three directions. The patient has an older brother who was diagnosed with autism, and the mother started to suspect that the patient also had autism. He was then referred for follow-up to his neurologist with the suspicion of autism.

At his two-year visit, the patient was diagnosed with ASD level 2 and could not stack eight cubes or wash and dry his hands. A referral to behavioral therapy was added to his ongoing therapies. The mother was proactive in making sure the child received physical, speech, and occupational therapy promptly, and improvement was noted, such as being able to ambulate. At his three-year visit, he could not balance and lift for 5 seconds, broad jump, copy an *O*, pedal a tricycle, or dress without supervision. At his four-year visit, the patient progressed to ASD level 2 with accompanying intellectual impairment requiring substantial support. He was saying single words, unable to button his shirt or say when he was cold, tired, and hungry. The patient was deemed to have speech and developmental delays. At his five-year visit, the patient had a continued gross developmental delay and is continuing all therapies; at this visit, he was noted to twirl around and exhibited hand wringing.

Patient D

Patient D's developmental journey has been marked by challenges and complex symptoms. At nine months of age, Patient D displayed significant developmental delays, including an inability to stand or sit without support and brief moments of standing. Gross motor skills were notably decreased at this stage. The patient's working differential diagnosis included cerebral palsy due to his severe motor delays. Because of this, the patient was referred to a geneticist and neurologist. By his first birthday, Patient D could not walk independently, signifying a profound delay in motor milestones. At 15 months, he continued to face difficulties, as he did not walk alone and struggled with self-feeding, although he could scribble spontaneously. At 18 months, his parents hesitated to pursue further diagnostic tests, likely due to community stereotyping. Patient D was observed to be able to dump raisins from a bottle and stack four cubes. His physical examination appeared normal except for decreased motor tone of his lower extremities. However, developmental concerns persisted as he did not follow simple commands, combine two different words, or stack eight cubes by his second birthday. His inability to draw a vertical line further emphasized his developmental delay, specifically fine motor delay. He was referred early on to physical therapy, occupational therapy, and later speech therapy, which he received with poor compliance.

At the age of four years, Patient D received a formal diagnosis of ASD at level 2, reflecting substantial challenges in social communication and the presence of repetitive behaviors. Additionally, he was diagnosed with a disorder of organic acid metabolism, expressive speech delay, global developmental delay (GDD), microcephaly, and neurological abnormalities. While a neurologist diagnosed GDD, Patient D started therapy initially but then discontinued and has not received therapy since 2022. He displayed some cognitive progress, recognizing his first name but not his last, and demonstrated the ability to draw a cross and identify the longer line. His comprehension skills were assessed at 3 out of 4, and he recognized colors at a 3 out of 4 level. Patient D continued to face difficulty separating from his mother and dressing without supervision, prompting consideration of possible fragile X syndrome and a recommendation for another neurology consultation. Prior genetic studies, including fragile X and microsomal array, were ordered, but parents refused to test. The patient was again referred for follow-up with genetics and a neurologist to convince parents of the need to test the patient. During examination, the patient still exhibits excessive temper tantrums and exaggerated crying. He is still very difficult to examine and often requires multiple providers when conducting his examinations. Although the diagnosis is established, the parents have not been consistent with taking his prescribed therapies.

Patient E

This case involves a six-year-old patient who presented with a history of developmental delays and notable deficits in various areas of development. The patient also had a medical history of infantile spasms treated with ACTH. The patient demonstrated gross motor delay with an inability to sit unassisted at his six-month exam and was referred to physical therapy. The patient's developmental history at 15 months displayed the inability to scribble spontaneously, which demonstrated delayed fine motor skills. By 18 months, developmental delays continued to be evident as the patient struggled with tasks such as dumping raisins, imitating actions, stacking cubes, pointing to body parts, and following directions. The patient was started early on with physical, occupational, and speech therapy. He also has a chromosome 19 abnormality. At the age of two years, the patient displayed some progress, including the ability to say *mama* and *dada*, babble, and stack eight cubes. However, developmental concerns were noted with the absence of two-word sentences and the inability to combine words. Furthermore, basic self-care tasks like washing and drying hands were challenging for the patient.

By the age of three years, the patient's developmental difficulties persisted, as evidenced by the inability to copy tasks and dress independently without supervision. However, there were some positive signs, such as the ability to balance on one foot.

At four years of age, the patient received a formal diagnosis of developmental delay, with specific emphasis on gross motor, fine motor, and expressive speech delays. The patient was also diagnosed with ASD at level 2, signifying significant challenges in social communication and repetitive behaviors; a referral to behavioral therapy was added to his therapies. Despite these challenges, the patient improved in certain areas, such as recognizing directions and colors, drawing simple shapes like a cross, and acknowledging basic needs like feeling cold, tired, or hungry. Additionally, the patient was observed to dress with supervision.

## Discussion

The purpose of this study is to present a case report looking at the clinical diagnosis approach of ASD for children younger than two years. Despite the advances in understanding and diagnosing ASD, the challenge remains to translate these findings into practical clinical applications that can be universally adopted across healthcare systems. This comprehensive study aims to set the stage for a case report that explores the clinical approach to diagnosing autism in children under two, explicitly focusing on gross/fine motor delay, chromosome 19 mutation, prematurity, macrocephaly, and false negative MCHAT.

One distinctive aspect of early autism diagnosis is the emphasis on gross motor developmental delay as a potential key indicator. The gross motor delay was observed in five out of the six selected cases; additionally, the fine motor delay was observed only in Patient A. Within these patients, the motor delays included the inability to walk unsupported by 15 months, stack two blocks at 15 months, stack eight cubes by two years, continuously scribble by 15 months, feed self, and dump raisins by 18 months. Some atypical presentations we saw across our case report were abnormal movements such as hand flapping, wringing, and twirling. In support of this observation, research conducted by Dominique et al. showed evidence of motor behaviors, general attention, and attention to social stimuli associated with children later diagnosed with ASD [2]. Similar findings were observed by Harris [8], who concluded that motor delays seen during the first year of life are a prodrome of ASD. Another prevalent comorbid condition commonly seen in children diagnosed with ASD is GDD. According to Shan et al., the prevalence of GDD in children with ASD was 62.3%, and the total average developmental quotient (DQ) of GDD was mildly deficient [9]. It was negatively correlated with the symptoms of autism, with a *P*-value less than 0.05. Within the realm of GDD, language appears to be severely compromised; similar findings are seen in patients with ASD. This factor has been known as a diagnostic criterion for diagnosing ASD, as evidenced by its assessment in the MCHAT.

MCHAT is a screening tool that has been used in the diagnosis of ASD. Among our case reports, two select patients (Patients A and B) initially presented with a negative MCHAT but were later diagnosed with ASD. Due to this frequently seen anomaly, many studies have been conducted that have investigated the validity of this screening tool. In a research study conducted by Guthrie et al., it was found that the sensitivity of the MCHAT was 38.8% with a PPV of 14.6%. However, sensitivity was higher in older toddlers [10]. The study concluded that the MCHAT was less accurate in detecting ASD than previous studies.

Interestingly, another investigative study compared the validity of the MCHAT-R (Modified Checklist for Autism in Toddlers Revised) with the original MCHAT. Robins et al. found that the MCHAT-R detected ASD at a higher rate compared to the original MCHAT while reducing the number of children requiring follow-up evaluations [11]. Moreover, children in this study were diagnosed with ASD at an average age of two years younger than the national median age of diagnosis. The study concluded that implementing universal screening with the MCHAT-R/F could significantly lower the age of ASD diagnosis by two years. Like the Robins study, Ramelli et al. also made a similar discovery in their study of a southern Switzerland population [12]. These findings help us better understand the false negative MCHAT that was prevalent in the observed cases from our study. 

Another study concluded that the overall diagnostic ability of the MCHAT in very preterm children was considered poor. This research study concluded a high false negative rate of 48% and a false positive rate of 16% [13]. The link between prematurity and autism has been a subject of interest in medical research. Some studies suggest a potential association between prematurity and an increased risk of ASD [14]. It is important to note that not all premature infants develop autism, and many factors contribute to the development of the disorder. However, prematurity can be a contributing factor, potentially due to immature brain development and neural connections in premature infants. Research indicates that premature birth may disrupt the normal course of brain development, particularly in regions associated with social communication and behavior regulation [15]. This disruption could contribute to the development of autism in some cases. The cases of Patients B and C align with these findings as the patients displayed delays in social communication, language acquisition, and motor skills - all areas commonly seen in premature children.

An interesting finding observed in both Patients C and E was the abnormality of chromosome 19, which was discovered at 18 months in both patients. Recent research showed a significant genetic finding of a de novo 1-Mb duplication involving 18 genes on Chromosome 19 [16]. This genetic variation is referred to as a copy-number variant (CNV), which represents an alteration in the number of copies of a specific DNA segment. The relevance of this CNV lies in the fact that within the affected family, there are multiple cases of neurodevelopmental disorders, including ASD, attention-deficit/hyperactivity disorder (ADHD), intellectual disability (ID), and psychiatric disease. This finding suggests a potential association between Chromosome 19 CNV and the occurrence of these neurodevelopmental disorders within the family. In the context of ASD, this Chromosome 19 CNV could be a critical genetic factor contributing to the occurrence of ASD within this family. Research has shown that ASD is a complex and heterogeneous disorder with a vital genetic component.

The case of Patient D sheds light on the complex challenges faced by individuals with ASD, particularly when associated with co-morbidities like macrocephaly. Patient D’s developmental journey was marked by early delays in motor skills and communications, which exemplifies the diversity within ASD diagnostic criteria. The reviewed literature reveals insight into the connection between macrocephaly and ASD [17]. While the majority of macrocephalic ASD cases lack a genetic causal link, historically, PTEN gene mutations have been linked to large head sizes in ASD patients study done by Klein et al [18]. estimated occurrence in approximately 22% of diagnosed cases. 

Another study by Hobert et al. saw 27% of PTEN mutations among macrocephalic ASD cases [19]. Additionally, they studied the prevalence of amino acid metabolism disorder among macrocephalic ASD cases. They observed an elevation of urine aspartic acid (87%), plasma taurine (69%), and reductions of plasma reduction of plasma cysteine (72%). Interestingly, in our case report, Patient D, diagnosed with macrocephaly, was also diagnosed with an amino acid metabolism disorder.

## Conclusions

This case report provides valuable insights into diagnosing ASD in children under two, highlighting the complexity and importance of considering various developmental milestones and factors. A significant finding is the prevalence of gross and fine motor delays, which are potential critical indicators for early ASD diagnosis. These motor delays and comorbid conditions like GDD underline the need for refined screening tools. The study also found limitations in MCHAT, as some patients initially had negative results but were later diagnosed with ASD. This discovery suggests the necessity for improved screening methods, such as the MCHAT-R, which shows promise for earlier detection.

The report further reveals the complexity of ASD presentations, noting the role of comorbidities like chromosome abnormalities, prematurity, and macrocephaly. A case with macrocephaly and an amino acid metabolism disorder highlights the heterogeneity of ASD, emphasizing the need for individualized assessments and interventions. Overall, the case report enhances understanding of early ASD diagnosis as well as stresses the importance of continued research and clinical vigilance in identifying and supporting children under two. Early intervention and tailored approaches are crucial for improving long-term outcomes and quality of life for individuals with ASD.
